# Design and Formative Evaluation of a Virtual Voice-Based Coach for Problem-solving Treatment: Observational Study

**DOI:** 10.2196/38092

**Published:** 2022-08-12

**Authors:** Thomas Kannampallil, Corina R Ronneberg, Nancy E Wittels, Vikas Kumar, Nan Lv, Joshua M Smyth, Ben S Gerber, Emily A Kringle, Jillian A Johnson, Philip Yu, Lesley E Steinman, Olu A Ajilore, Jun Ma

**Affiliations:** 1 Washington University School of Medicine St. Louis, MO United States; 2 University of Illinois at Chicago Chicago, IL United States; 3 The Pennsylvania State University University Park, PA United States; 4 University of Massachusetts Medical School Worcester, MA United States; 5 University of Washington Seattle, WA United States

**Keywords:** voice assistants, behavioral therapy, problem-solving therapy, mental health, artificial intelligence, user evaluation

## Abstract

**Background:**

Artificial intelligence has provided new opportunities for human interactions with technology for the practice of medicine. Among the recent artificial intelligence innovations, personal voice assistants have been broadly adopted. This highlights their potential for health care–related applications such as behavioral counseling to promote healthy lifestyle habits and emotional well-being. However, the use of voice-based applications for behavioral therapy has not been previously evaluated.

**Objective:**

This study aimed to conduct a formative user evaluation of Lumen, a virtual voice-based coach developed as an Alexa skill that delivers evidence-based, problem-solving treatment for patients with mild to moderate depression and/or anxiety.

**Methods:**

A total of 26 participants completed 2 therapy sessions—an introductory (session 1) and a problem-solving (session 2)—with Lumen. Following each session with Lumen, participants completed user experience, task-related workload, and work alliance surveys. They also participated in semistructured interviews addressing the benefits, challenges and barriers to Lumen use, and design recommendations. We evaluated the differences in user experience, task load, and work alliance between sessions using 2-tailed paired *t* tests. Interview transcripts were coded using an inductive thematic analysis to characterize the participants’ perspectives regarding Lumen use.

**Results:**

Participants found Lumen to provide high pragmatic usability and favorable user experience, with marginal task load during interactions for both Lumen sessions. However, participants experienced a higher temporal workload during the problem-solving session, suggesting a feeling of being rushed during their communicative interactions. On the basis of the qualitative analysis, the following themes were identified: Lumen’s on-demand accessibility and the delivery of a complex problem-solving treatment task with a simplistic structure for achieving therapy goals; themes related to Lumen improvements included streamlining and improved personalization of conversations, slower pacing of conversations, and providing additional context during therapy sessions.

**Conclusions:**

On the basis of an in-depth formative evaluation, we found that Lumen supported the ability to conduct cognitively plausible interactions for the delivery of behavioral therapy. Several design suggestions identified from the study including reducing temporal and cognitive load during conversational interactions, developing more natural conversations, and expanding privacy and security features were incorporated in the revised version of Lumen. Although further research is needed, the promising findings from this study highlight the potential for using Lumen to deliver personalized and accessible mental health care, filling a gap in traditional mental health services.

## Introduction

Artificial intelligence (AI) has provided new opportunities for human interactions with technology for care delivery [1]. These include remote monitoring, mobile health apps (eg, chatbots), and the use of a wide variety of sensors for remote monitoring and surveillance. Of the recent innovations, personal voice assistants that rely on AI-based platforms such as Amazon’s Alexa, Google Home, Cortana, and Siri have transformed how humans search for information, with recent reports suggesting that nearly 30% of search queries rely on voice-based input [2]. Broad adoption of such platforms lends support for their potential utility in health care–related applications such as behavioral counseling to promote healthy lifestyle habits and emotional well-being [3,4]. However, current health care–related applications of voice assistants are generally rudimentary, and few of them have been developed for delivering evidence-based therapies or have been subjected to careful evaluation (eg, to inform development or for their effect on clinical or behavioral outcomes) [5]. To this end, we developed and evaluated Lumen, an end-to-end voice-based virtual coach that was developed as a stand-alone Alexa application. Lumen delivers evidence-based problem-solving treatment (PST) for patients with mild to moderate symptoms of depression and anxiety.

Lumen, by design, is different from the current spectrum of voice-based health applications that primarily support web-based information–seeking activities [4]. Studies on such information-seeking activities performed on voice assistants have focused on the quality and content of voice assistant responses for several topics including health behavior and lifestyle [6,7], mental health, interpersonal violence, addiction help [8,9], patient and consumer safety risks [10], vaccines [11], postpartum depression [12], medication names [13], and sexual health [14]. The findings across these studies consistently highlight the shortcomings associated with the quality of the information retrieved during these voice-based searches. For example, Bickmore et al [10] found that Siri, Alexa, and Google Assistant platforms and their underlying algorithms were effective in completing only 43% of requests regarding situations that required medical expertise, and 29% of the responses could have resulted in some degree of patient harm [10]. Other applications, mostly preliminary prototypes, have been developed for assessment and support. These applications have been used for delivering visual acuity tests [15], support for coping with chronic disease [16], and for nutritional planning [17]. However, it is important to note that these applications have largely lacked outcome assessment or incorporation of behavioral therapy [4]. Although text-based behavioral therapy applications (eg, chatbots) have shown promise in mitigating psychiatric disorders [18,19], several challenges exist including long-term adherence and engagement limited to younger age groups [20]. Therefore, it is plausible that voice-based therapy delivery may mitigate some of these issues.

In this paper, we describe the design and formative evaluation of Lumen, with the following research objectives: (1) to characterize the user experience, task-related workload associated with interactive communication, and participant alliance with delivered treatment and (2) to identify and describe user perspectives including the benefits, challenges, and barriers to Lumen use and recommendations for design improvements.

## Methods

In the following sections, we describe the design components of Lumen, its features, and the mixed methods study that was conducted.

### Lumen

Lumen is a virtual voice-based coach that delivers an evidence-based, 8-session PST program for patients with mild to moderate depression and anxiety. The first 4 PST sessions were conducted weekly, followed by 4 biweekly sessions. Each PST session lasted approximately 45 minutes to 1 hour. Lumen was designed to align with the evidence-based PST program.

**Figure 1:**
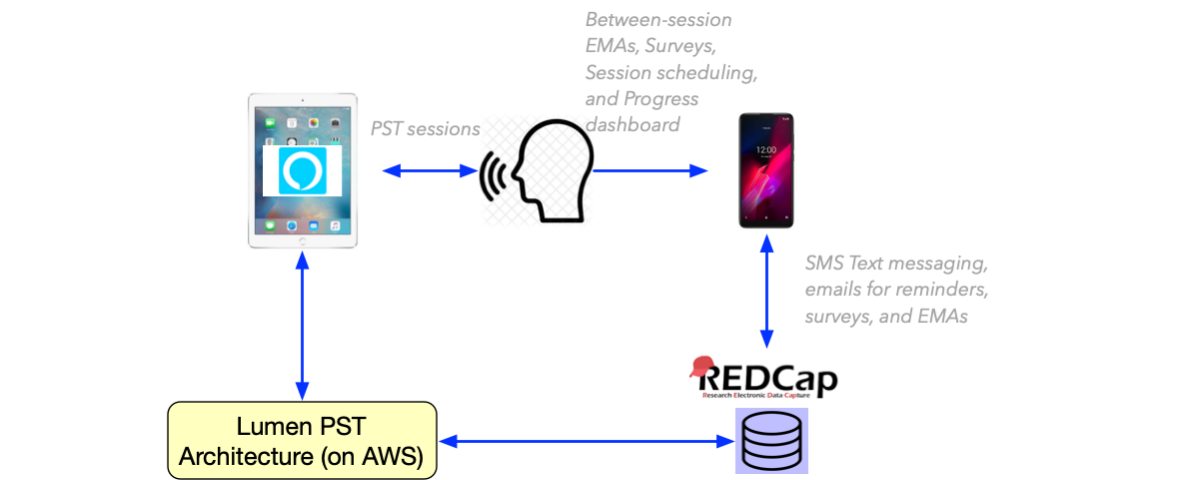
User interaction with Lumen for problem-solving treatment (PST) sessions highlighting the various components. AWS: Amazon Web Services; EMA: ecological momentary assessment.

Lumen’s design was based on two overarching principles: (1) providing cognitively plausible conversations, that is, aligning Lumen’s conversations with the cognitive processes of human communicative interactions [5] and (2) alignment with the principles of evidence-based PST. This PST program was previously tested and delivered with a human coach [21]; Lumen incorporates essential components of the treatment protocol for coaching and monitors progress using surveys and ecological momentary assessments. All Lumen design components are delivered in an integrated environment, coordinated through the voice-only platform and associated mobile tools (Figure 1 provides an overview of the components of Lumen and their interactions).

Developed on Amazon’s Alexa platform, Lumen’s architecture incorporates an intelligent conversation manager that manages the content, structure, and flow of interactive conversations between a patient and Lumen and a context manager that incorporates context awareness into the conversations. Using underlying AI capabilities of the Alexa platform, the conversation manager uses user verbal input to provide appropriate, synchronous responses, aligned with PST’s treatment guidelines. PST content and conversational structure were designed in consultation with master PST trainers and PST experts.

The context manager provides contextual awareness to the interactions by incorporating user input from surveys and ecological momentary assessments (delivered asynchronously through mobile apps) and treatment progression and continuity (eg, review of patient problems and action plans from a previous session; Sections A and B in Multimedia Appendix 1 provide additional details of the Lumen architecture and features).

We followed an iterative user-centered design process, comprising brainstorming sessions with software engineers, interaction designers, psychiatrists, and researchers; prototype development on the Alexa platform; and several iterations of internal testing.

### Participants and Study Design

Participants for this formative evaluation were recruited from the recently completed Engaging Self-Regulation Targets to Understand the Mechanisms of Behavior Change and Improve Mood and Weight Outcomes (ENGAGE-2) trial (ClinicalTrials.gov, National Clinical Trial#03841682), in which a PST-certified health coach delivered integrated collaborative care for depression and obesity to intervention participants, whereas those in the control group received usual care. A convenience sample (91/106, 85.8%) of ENGAGE-2 participants was contacted for assessing their interest in participating in a study with a virtual PST coach. Of these 91 participants, 26 (28%) expressed interest and consented to participate. Of the 26 participants, 17 (65%) had prior PST experience (ie, part of the ENGAGE-2 intervention group) and 9 (35%) did not have prior PST experience (ie, part of the ENGAGE-2 control group).

This was an observational study, with each participant completing 2 Lumen sessions: an *introductory* first session (termed S1; n=26) and a *problem-solving* second session (termed S2; n=24, missing 2 of the 9 ENGAGE-2 control participants). The 2 sessions represented the overarching structure of the 8-session, evidence-based PST evaluated in a previous trial [21]; S1 represented an initial overview session, and S2 represented a problem-solving session that was repeated in sessions 2 to 8 during the evidence-based PST.

In S1, Lumen provides a program overview, provides a detailed introduction to the PST process and behavioral activation, and guides the participant to create a list of problems to address in subsequent sessions. In S2, Lumen guides the participant through the steps of problem-solving: identifying a problem to address, setting a goal, brainstorming possible solutions, evaluating the pros and cons of each solution, selecting a solution to implement, and developing an action plan to carry out before the next session. S2 concludes with behavioral activation coaching, where Lumen assists participants with selecting a social, physical, and pleasant activity to partake before the next session.

The full Lumen PST program included 6 more problem-solving sessions that followed the same structure as S2; this was the rationale for testing only 1 problem-solving session during this formative evaluation. As such, the purpose of the 2-session approach was to conduct a representative evaluation of all Lumen sessions and to evaluate whether there were differences in participant experience and interactions between the sessions.

### Ethics Approval

The study was approved by the institutional review board of the University of Illinois (IRB#2020-0918). All participants provided written consent.

### Procedure

Consented participants were provided access to the Lumen S1 and S2 skills via the Alexa application and were given instructions on how to enable the skills on the Alexa app on their personal phones or mobile devices. All user interviews were conducted remotely by a trained research coordinator using the Zoom (Zoom Video Communications) videoconferencing platform. Participants were first provided with a brief overview of the study purpose, and their access to the Lumen skill (designed as a private skill, which was available by invitation only) was verified. During the session, a research coordinator went through a list of tips to effectively communicate with Lumen and answer any questions. After this, participants were instructed to turn off their video, and audio recording via Zoom was enabled from this point. Participants then opened the Alexa app and said “Open Lumen Coach” to begin their Lumen session. During their Lumen sessions, the trained note taker took notes of any deviations from the session script or any technical problems.

After each Lumen session, the coordinator followed a semistructured interview script that included the following components. First, participants were asked to walk through their interaction experience with Lumen during their completed session, reflecting on what worked, what did not, and challenges they faced. Although the same procedure was followed for both Lumen sessions, interview questions varied slightly from S1 to S2 to inquire about session-specific content. Interview questions after S1 focused on participants’ impressions of Lumen, suggestions for improving Lumen, evaluating the usefulness of tips on how to communicate with Lumen, and impressions of the PST overview. Interview questions after S2 included questions about participants’ impressions of Lumen that were different from S1, delivery of PST by Lumen, and factors affecting their likelihood of Lumen use in the future. S1 and S2 were conducted several days apart, and participants had access to the specific sessions only a day or so before the session.

After the interviews were completed, participants were emailed a link to 3 brief postinterview surveys related to user experience, workload, and the collaborative relationship between the participant and Lumen (User Experience Questionnaire Short Version [UEQ-S] [22], NASA Task Load Index [TLX] [23], and Working Alliance Inventory–Technology Version [WAI-Tech] [24]).

Audio recordings of the interviews (26 for S1 and 24 for S2) were transcribed using the Trint audio transcription software for subsequent analysis. All (26/26, 100%) postinterview surveys were completed after S1, and 95% (23/24) postinterview surveys were completed after S2.

### Data Analysis

Data analysis included coding of interview transcripts using thematic analyses and descriptive summaries of user experience, task load, and WAI-Tech surveys.

#### Coding of Transcripts

All interview transcripts were coded using an inductive thematic analysis to characterize the participants’ perspectives regarding their interaction with Lumen [25] (Section E in Multimedia Appendix 1 provides the interview guide). This approach involved the following stages: first, 2 coauthors (CRR and EAK) read the interview transcripts to familiarize themselves with the content. Next, a set of “open codes” was created to characterize the content and context discussed in the interviews (ie, inductive coding) [26]. These initial codes were compared across the transcripts to identify repeated and interrelated subthemes. Similar subthemes were grouped over multiple review sessions to develop a set of 6 overarching themes. All responses were coded; some responses were assigned multiple codes, in an order of relevance; however, only the primary assigned code was used for all analyses. Two coauthors (EAK and CRR) independently coded a set of 5 transcripts with a high degree of interrater agreement (Cohen κ ranged from 0.83 to 1 with mean 0.93, SD 0.07). Discrepancies were resolved through discussions with the first author (TK). Subsequently, all remaining transcripts were coded.

#### Surveys

From the UEQ-S survey, pragmatic quality and hedonic quality scale values were calculated by rescaling the survey responses to the range −3 to 3 and calculating item means within each scale using the UEQ-S Data Analysis Tool [27]. Pragmatic quality refers to the task- or goal-related interaction qualities (eg, efficiency, perspicuity, and dependability) that a user aims to reach when using the product. Hedonic quality refers to the aspects related to pleasure or fun (eg, stimulation and novelty) while using the product. Values <−0.8 represent a negative evaluation, between −0.8 and 0.8 represent a neutral evaluation, and >0.8 represent a positive evaluation on each scale.

The NASA TLX rating sheet was administered assuming similar weights for each of the 5 task load items (except for physical demand, which was not considered, as it was irrelevant to Lumen): mental demand, temporal demand (eg, being rushed), effort, frustration, and performance. Each item was then rescaled to the range 5 to 100 by multiplying the raw score by 5.

From the WAI-Tech survey, three 12-item subscale (task, goal, and bond) scores and an overall score were calculated as item means within each subscale. The task subscale reflected how responsive Lumen was to the participant’s focus or need; the goal subscale reflected the extent to which goals were important, mutual, and capable of being accomplished; and the bond subscale reflected the degree of mutual liking and attachment [24]. A higher overall score reflected a more positive rating of the working alliance.

Given that the 2 sessions focused on 2 primary structural components of PST sessions—a session overview and a problem-solving session—we compared whether there were differences in the user experience, task load, or work alliance between these sessions. To this end, scores on each of the scales between S1 and S2 were compared using paired *t* tests. Analyses were conducted using SAS (version 9.4; SAS Institute Inc); statistical significance was defined by 2-sided *P*<.05. Additional analyses comparing PST-experienced and PST-naive participants can be found in Section F in Multimedia Appendix 1.

## Results

### General Characteristics

Among the 26 participants, 20 (77%) were female, 19 (73%) were racial or ethnic minorities (n=13, 50% Black; n=6, 23% Hispanic) with an average age of 43.9 (SD 11.9) years, 10 (38%) had a high school or some college education, and 14 (54%) had an annual family income of <US $55,000 (Table 1). Participants with previous PST experience (17/26, 65%) and those without previous PST experience (9/26, 35%) did not differ in age, race, income, or educational status, although 65% (11/17) of the participants with previous PST experience and 100% (9/9) of the participants without PST experience were female (*P*=.04).

**Table 1:** Baseline characteristics by prior problem-solving treatment (PST) experience.

Characteristic		All Lumen formative evaluation participants (N=26)	Participants with prior PST experience (n=17)	Participants without prior PST experience (n=9)	*P* value
Age (years), mean (SD)		43.9 (11.9)	42.6 (13.2)	46.3 (9.2)	.46
Female, n (%)		20 (77)	11 (65)	9 (100)	.04
**Race or ethnicity, n (%)**					
	Non-Hispanic White	4 (15)	3 (18)	1 (11)	.34
	African American	13 (50)	9 (53)	4 (44)	.34
	Asian or Pacific Islander	1 (4)	1 (6)	0 (0)	.34
	Hispanic	6 (23)	2 (12)	4 (44)	.34
	Other (eg, decline to state or multirace)	2 (8)	2 (12)	0 (0)	.34
**Education, n (%)**					
	High school or general education or less	2 (8)	1 (6)	1 (11)	.95
	College—1 year to 3 years	8 (31)	5 (29)	3 (33)	.95
	College—≥4 years	10 (38)	7 (41)	3 (33)	.95
	Post college	6 (23)	4 (23)	2 (22)	.95
**Income (US $), n (%)**					
	<35,000	7 (27)	4 (23)	3 (33)	.32
	35,000 to <55,000	7 (27)	3 (18)	4 (44)	.32
	55,000 to <75,000	5 (19)	4 (23)	1 (11)	.32
	≥75,000	7 (27)	6 (35)	1 (11)	.32

### User Experience, Task Load, and Working Alliance

Participants had a positive evaluation (values >0.8) for pragmatic (S1: mean 1.3, SD 1.2 and S2: mean 1.4, SD 0.9), hedonic (S1: mean 1.0, SD 1.1; S2: mean 1.2, SD 1.0), and overall (S1: mean 1.2, SD 1.0; S2: mean 1.3, SD 0.8) qualities related to their user experience with Lumen for both sessions. There were no statistically significant differences between the 2 sessions (t_22_=0.37, 0.00, and 0.25 and *P*=.71, .99, and .80 for pragmatic, hedonic, and overall scores, respectively).

Across both sessions, participants encountered medium (approximately 50) across the mental (cognitive), effort, frustration, and performance dimensions of the NASA TLX scale. There were no statistically significant differences between S1 and S2 (Table 2). However, participants rated as having experienced more temporal workload in S2 (mean 52.0, SD 29.1) than S1 (mean 36.5, SD 23.2; *P*=.03), suggesting feeling rushed during their interaction with Lumen in S2.

The scores on the 7-point WAI-Tech survey for task (S1: mean 5.2, SD 0.9; S2: mean 5.3, SD 0.9), bond (S1: mean 4.9, SD 1.0; S2: mean 4.7, SD 1.0), and goal (S1: mean 5.0, SD 0.9; S2: mean 5.1, SD 0.9) subscales were moderately high, indicating that Lumen-based PST sessions were perceived to be aligned with the participants’ needs, addressing their potential goals and the degree of mutual liking. There were no statistically significant differences between both sessions on the task, goal, and bond scales or the overall scores (Table 3).

**Table 2:** Paired *t* test results comparing NASA Task Load Index scores between sessions 1 and 2.

Question	Session 1 (n=26), mean (SD)	Session 2, (n=23), mean (SD)	*t* test (*df*)	*P* value
How mentally demanding was the task? (*mental demand*^a^)	42.7 (25.0)	53.9 (26.1)	−1.80 (22)	.09
How hurried or rushed were you in the pace of the task? (*temporal demand*)	36.5 (23.2)	52.0 (29.1)	−2.37 (22)	.03
How hard did you have to work to accomplish your level of performance? (*effort*)	36.0 (23.4)	42.8 (18.9)	−1.44 (22)	.16
How insecure, discouraged, irritated, stressed, and annoyed were you? (*frustration*)	31.9 (22.0)	38.5 (24.6)	−0.95 (22)	.35
How successful were you in accomplishing what you were asked to do? (*performance*)	34.6 (23.1)	37.2 (23.3)	−0.37 (22)	.71

^a^Italicized text shows the various categories of the NASA Task Load Index scales.

**Table 3:** Paired *t* test results comparing task, goal, and bond subscales of the Working Alliance Inventory–Technology Version between sessions 1 and 2.

Scale	Session 1, mean (SD)	Session 2, mean (SD)	*t* test (*df*)	*P* value
Task subscale	5.2 (0.9)	5.3 (0.9)	0.11 (22)	.92
Bond subscale	4.9 (1.0)	4.7 (1.0)	1.49 (22)	.15
Goal subscale	5.0 (0.9)	5.1 (0.9)	−0.32 (22)	.75
Overall scale	5.0 (0.9)	5.0 (0.9)	0.56 (22)	.58

### User Perspectives of Lumen

On the basis of the thematic analysis, we identified 6 categories that highlighted key user perspectives regarding Lumen. This included (total, N=536 coded themes across all categories; % of each category across all transcripts): (1) comparing Lumen with a human coach (ie, a human-AI comparison; 200/536, 37.3%), (2) task load experienced during Lumen interactions (102/536, 19%), (3) perception of PST delivered by Lumen (82/536, 15.2%), (4) user suggestions for improving Lumen (81/536, 15.1%), (5) natural language understanding of Lumen (44/536, 8.2%), and (6) technical issues (27/536, 5%) that were encountered during the 2 Lumen sessions (detailed descriptions of each of these categories along with exemplary quotations are provided in Table 4).

Comparisons of Lumen with a human coach included several aspects: potential flexibility, ease of accessibility of Lumen for those who cannot attend face-to-face appointments, and cost-related advantages. Participants also highlighted the nonhuman nature of the interaction, describing the lack of changes in tone, emotion, instant feedback, and desiring a “more personalized human touch.” Nevertheless, nearly all participants described the potential advantages related to Lumen’s accessibility, allowing those in need for therapy easily access a coach at any time:

...the fact that the flexibility of it, the fact that I could be at home, where I could be in my car, or that, you know, I could take a moment and stop at work and go in a quiet room instead of having to, you know, actually go out and, you know, go to a building, find parking, all of the inconveniences that come with [face-to-face] appointments...

In addition, and importantly, participants with previous PST experience expressed that the Lumen sessions were similar to the human coach sessions that they had previously engaged in.

Participants also highlighted the workload associated with Lumen sessions, sometimes describing the difficulty in pausing sessions to collect thoughts as they worked through the steps of PST. This was especially the case in S2, where participants were required to brainstorm multiple solutions to a problem and then list the pros and cons of each solution. The workload challenges identified were related to pacing of the sessions (temporal load) and the amount of information that was directed at the participants (cognitive load). One of the participants explained that the short time to respond made them “feel pressured to come up with something ...[...]. But she [Lumen] did ask if I needed more time, but when I was responding my answers, I [still] felt like it was a short time and I almost felt cut off*.*”

Participants described their perceptions of the PST program or structure as well as Lumen’s role in delivering PST. Their comments highlighted the importance of the PST stepwise structured approach and Lumen’s PST coaching that enabled them to create goals that could have been overwhelming:

If my goal is truly trying and I have a problem, I just feel overwhelmed. I don’t know how to attack it. Well, Lumen supplies that. It breaks it down. It pulls all of the jumbled information out of my head, leaves the emotion behind and helps me lay out a plan for essentially attacking the problem without the emotional stress of it.

Participants provided several suggestions for improvement. This included further personalizing the PST sessions, creating embodied avatars for Lumen, incorporating a friendlier voice, and investigating ways for reducing the task load associated with the interactions. One of the most insightful aspects was several participants highlighting the importance of cognitive “offloading” [28]. This was especially aligned with the need to reduce the cognitive load associated with conversational interactions, especially during the problem-solving session (S2), where participants had to identify and work through a problem, set a goal, identify and evaluate possible solutions, and then devise a structured action plan to address the problem. Participants also suggested the need for visualizing their tasks, either digital or paper-based, that would help in organizing their thought processes and saving the notes for future interactions, as highlighted in the following quote: “*If it would have a way in app, I mean, [...] but like a way to help me, a way to help track for me what my progress is.*”

Although there were a few instances of technical issues where the participants’ verbal responses were not comprehended by Lumen because of issues related to accent or ambient noise, these issues were minimal and most users noted the ease of interaction, as described in the following quote: “I was pretty much impressed with how easy was to use and, you know, it wasn’t intimidating at all*.*” Additional examples of Lumen interactions including problem-solving conversations are provided in Section D in Multimedia Appendix 1.

**Table 4:** Coding categories, their description, and examples from the interviews.

Coding category (spread^a^, %)	Description	Example from data
Interactive task load (78%)	Participant description of the demands of interacting with Lumen. Includes: Temporal load (pace of interactions, whether there was ample time to provide a response) Cognitive load (density of content and length of sessions)	“I felt kind of rushed when it was like time to, like, think through and write things” (3502) [Temporal load] “Sometimes it’s telling you a lot of things. So, for a user, it’s hard...You’re not looking at somebody. So, you’re really, really having to concentrate and pay attention, so if by any chance you miss something, then you kind of get lost” (1213) [Cognitive load]
Natural language understanding (46%)	Participant description of challenges that Lumen faced with understanding participants’ verbal responses. Includes: Spoken comprehension (breakdowns due to Lumen’s comprehension) Accent or enunciation issues (eg, understanding names)	“I think it was difficult to provide the prompts that were requested, and I suspect that depending on the person’s accent or if they’re from—if maybe their English isn’t exactly clear, there may be some language issues” (5457) [Spoken comprehension and accent or enunciation issues]
Comparison with human coach (100%)	Comparison of Lumen to a human coach. Includes: Naturalness of voice or tone (presence or absence of emotion) Interactive engagement in conversation (whether Lumen was conversational) Lumen’s tone or inflection (identifying when Lumen was asking a question vs making a statement) Lumen vs human PST^b^ content (comparing depth of help Lumen provided relative to human in delivery of PST) Perceived Lumen benefits or drawbacks (pros and cons of receiving PST from Lumen relative to human, eg, accessibility, availability, and comfort with disclosure)	“...just robotic. Like, I’m talking to like a machine robot. That’s my initial thought. But at the same time, not in the way that it’s like dumb, but in that it’s like very scientific and not very like human.” (6132) (Naturalness of voice or tone) “I think initially for me, what may be missing that I picked up on right away is the human interaction component. [...] a human as opposed to talking to like a device or a computer [...] So, I don’t know how differently it'll be the more I become engaged with it.” (3498) [Interactive engagement in conversation] “When I spoke with [the human coach], I found myself venting, if I may, and going in every which direction, whereas Lumen forces me to stay very rigid, and sometimes when going through problem solving, the emotional release of going in every which direction, direction, rather than going straight and narrow feels a lot more comfortable.” (3831) [Lumen vs human PST] “it allows accessibility to people who can’t travel or maybe they feel anxious around talking to another person. So, it eliminates like class, it eliminates race, it eliminates sex. It eliminates sort of those prejudice that could happen in like a person-to-person to person setting.” (6132) [Perceived Lumen benefits]
PST features in Lumen (78%)	Description of the PST features as delivered by Lumen. Includes: Program structure or format (feedback around the stepwise PST process) Virtual PST coaching (describing Lumen’s role in the PST process)	“You know, I think if I’m if I am if my goal is truly trying and I have a problem, I just feel overwhelmed. I don’t know how to attack it. Well Lumen supplies that. It breaks it down. It pulls all of the jumbled information out of my head, leaves the emotion behind and helps me lay out a plan for essentially attacking the problem without the emotional stress of it.” (3831) [Program structure or format and virtual PST coaching]
User recommendations (62%)	Participants’ recommendations for: Lumen improvements (ideas for functions or features in the user interface) Interacting with Lumen (tips for others to have an effective session with Lumen)	“I would tell them that like, so like you’re talking to a computerized app, so make sure you’re speaking clearly and slowly and like follow directions in order to get what you’re what you need from it.” (6132) [Interacting with Lumen] “I would say as a part of the app, have basically have the binder already inside the app and then maybe have a link to a principal PDF for those who want to do that.” (6023) [Lumen improvements] “I think it would be kind of cool, especially with it being linked with Alexa is if it had the ability to pick up keywords. So, like if I, you know, saying like I need to work on my diet or trainer or whatever, that somehow it was able to tap into some of those keywords. And while it’s talking back to me saying, you know. You know, we’ve looked into like some trainings in your area. We are going to send you emails of, you know, something like that that would be like really great or hear from information regarding blah, blah, blah, blah, blah.” (3498) [Lumen improvements] “She could be better if she if I could see it, even though is a mechanical thing or robot, I want to see Lumen, so I know how Lumen it looks...I’d rather see the person I’m talking to, even though [it] is a machine or whatever it is I would rather see, you know.” (7323) [Lumen improvements]
Technical issues (36%)	Technical issues that were experienced by participants during the sessions. Includes: Breakdowns in conversation	“Well, I was a little confused when it just stopped. It was still on the app. [...] And then it just completely shut the app.” (3470) [Breakdowns in conversation]

^a^Spread refers to the percentage of transcripts (total=50) that the coding category was present.

^b^PST: problem-solving treatment.

## Discussion

### Principal Findings

We designed and developed a virtual voice-based coach, Lumen, which delivers an evidence-based PST program for depression and anxiety. To the best of our knowledge, Lumen is one of the first voice-based virtual coach application for delivering behavioral therapy. In contrast to prior research that has primarily used voice assistants in web-based information–seeking tasks, Lumen delivers therapy aligned with the goals and principles of an empirically validated PST program. In this developmental evaluation, participants found the Lumen virtual coach to have high pragmatic usability and user experience, with limited task load during interactions. Participants also highlighted the considerable advantages of Lumen including the on-demand accessibility to a virtual therapist and the delivery of a complex PST task with a simplistic structure and organization for achieving therapy goals. Moreover, although the second session required increased user input, there were no marked differences in effort or interaction quality, except for temporal load (associated with the pace of the conversations), which was highlighted by the participants in their interviews. In addition, the participants highlighted the lack of personalization and deep engagement in the conversation and the relative lack of emotional engagement in the conversations.

### Comparison With Prior Work

PST, traditionally delivered by human coaches in face-to-face or phone-based settings, has been developed on mobile platforms [29]. However, similar to other text-based mobile apps, participant engagement with mobile PST platforms has been challenging [30]. To this end, Lumen offers a novel, voice-based mechanism for seemingly naturalistic voice interactions, potentially replicating interactions with a therapist. As previously described, much of the prior work has relied on evaluating the quality health information–seeking tasks using voice-based personal assistants (eg, [8,9]). Moreover, many of the previously developed applications have been preliminary prototypes (eg, [15]) that lacked extensive evaluation or outcome assessment. To the best of our knowledge, this is one of the first fully functional voice-based applications that provides end-to-end support for behavioral therapy (in this case, PST).

### Design Changes

Several design changes were incorporated in response to participants’ suggestions. To reduce the temporal and cognitive load (ie, reducing the pace of conversations), we incorporated multiple functionalities within Lumen. First, we split longer conversations (especially in S1, where Lumen provided an overview of the PST) into multiple shorter conversations to reduce the mean length of conversations between Lumen and the participant. Such shorter conversations allow for more interactive turns and have been shown to improve the common ground and engagement between conversational partners [5,31-34]. Second, we developed functionality that allowed participants to repeat, pause, and resume conversations. This allowed participants to ask Lumen to repeat instructions if they could not keep up with the content or to pause conversations in situations where they needed to take a break. Finally, we slowed the pace of the conversations to reduce temporal demand.

In addition, based on suggestions, we also developed a workbook to accompany Lumen in both physical and digital forms. The workbook includes content corresponding to the PST and simple worksheets for taking notes and facilitating brainstorming problem-solving goals, developing and evaluating potential solutions, and creating action plans. Such a cognitive aid helps in externalizing the thought processes [28,35,36] and creating a record for follow-up after the session. Recording and brainstorming with tools also affords cognitive benefits, especially with older adults, such as prospective memory regarding the goals and action plans that were created [37]. We also developed several features linked to Lumen to further integrate contextual aspects regarding the user including their current status and progress. For example, participants can track their progress by viewing their completed sessions and responses to the Patient Health Questionnaire-9 and General Anxiety Disorder-7 surveys on a user dashboard. Similarly, responses on the Patient Health Questionnaire-9 and General Anxiety Disorder-7 surveys were integrated into the Lumen session and reviewed during the session to help participants monitor the level of their depressive and anxiety symptoms.

Finally, we heeded several privacy and security considerations for pragmatic implementation and testing in a real-world setting within the context of a planned pilot randomized clinical trial. To this end, we will afford trial participants access to the Lumen skill within the Amazon Alexa app on a fully encrypted and locked down iPad, with timed exits for nonuse. This allows for preventing accidental recording issues that have been reported regarding the use of voice-based smart devices (Section B, Multimedia Appendix 1). The iPad-based delivery is aligned with the concept of using a stand-alone “device as a therapist” for the planned trial. However, additional considerations regarding voice-based profile verifications and security considerations are necessary for a wider, pragmatic use of Lumen as a daily therapy tool.

Despite these changes, several aspects of Lumen’s design and interaction are limited by current AI-based voice technology. In particular, the natural language understanding challenges of voice-based technology are well documented [10]. These include difficulties in parsing tone, accent, and pronunciation in spoken language, creating breakdowns in conversation and making it functionally impossible to have a free-form, open-ended conversation with these devices. In addition, current technology is also not able to discern differences in emotion or other verbal cues that are easily interpreted in face-to-face human conversations [5]. With ongoing improvements in technology, these challenges are likely to be mitigated over time, allowing for continued improvement of Lumen for optimized user experience.

### Limitations

This mixed methods formative research study had several limitations. The study was based on a small sample of users (N=26) who used Lumen in a relatively controlled environment. However, participants were engaged in 2 sessions and performed the Lumen interactions without external support. Only 2 sessions were evaluated with participants, and as such, we could not characterize participants’ experience with the entire 8-session PST program. However, structurally, sessions 2 to 8 mirror the S2 evaluated in this study. It is likely that participants will become more or less comfortable with the Lumen interactions in the later sessions. Given the formative and controlled nature of this study, we could not assess the impact of the various measures (ie, task load and work alliance) over time. We will be able to determine such longitudinal effects in our ongoing pilot clinical trial. Sessions were attended by a research coordinator and a trained note taker. It is not known whether their presence influenced the participants’ use of Lumen or their responses to the interview questions.

Notwithstanding those technological and research limitations, the findings from the formative evaluation and the subsequent improvements in design and functionalities position Lumen to be a “minimum viable product” that is highly acceptable to participants, appears to veridically reflect PST content, and is ready for potential real-world pilot testing. Recruitment has been completed for the pilot clinical trial (ClinicalTrials.gov, NCT# 04524104) in which 63 adults with mild to moderate depressive and anxiety symptoms have been randomized in a 2:1 ratio to the Lumen intervention or the wait-list control group and followed for 4 months. The objectives of the pilot trial are 3-fold: (1) to determine the feasibility and acceptability of the Lumen virtual coach for delivering the 8-session PST program; (2) to assess neural target engagement by comparing changes in the amygdala and dorsal lateral prefrontal cortex in functional neuroimaging between the Lumen intervention and wait-list control groups; and (3) to examine the relationship between neural target engagement and changes in self-reported measures of mood, coping, and psychosocial functioning. The pilot trial will provide the preliminary data needed to accelerate the clinical and translational research on this novel digital psychotherapy and to catalyze future development and definitive efficacy clinical trials.

### Conclusions

With a goal of overcoming the lack of empirical evidence for AI-based voice applications in behavioral therapy, we developed a voice-only virtual coach, Lumen, for delivering PST. The findings from the formative evaluation highlight feasibility, accessibility, and favorable user experience. Suggestions for more natural conversations and better contextual support have resulted in an improved, minimally viable product. Lumen is being tested in a clinical trial to evaluate its neural mechanism of action and therapeutic potential in depression and anxiety. If successful, Lumen can be a viable voice-based therapist offering a realistic and cognitively plausible verbal interaction for personalized and accessible mental health care, filling a gap in traditional mental health services.

## Appendix

Multimedia Appendix 1 Details of the Lumen architecture and user interaction patterns with Lumen.

## Abbreviations

AI: artificial intelligence
ENGAGE-2: Engaging Self-Regulation Targets to Understand the Mechanisms of Behavior Change and Improve Mood and Weight Outcomes
PST: problem-solving treatment
TLX: Task Load Index
UEQ-S: User Experience Questionnaire Short Version
WAI-Tech: Working Alliance Inventory–Technology Version

## Data Availability

Deidentified data from this study are not available in a public archive. Deidentified data from this study will be made available (as permissible according to institutional review board standards) by emailing the corresponding author.
